# Longitudinal changes and determinants of parental willingness to pay for the prevention of childhood overweight and obesity

**DOI:** 10.1186/s13561-020-00266-z

**Published:** 2020-05-28

**Authors:** Romy Lauer, Meike Traub, Sylvia Hansen, Reinhold Kilian, Jürgen Michael Steinacker, Dorothea Kesztyüs

**Affiliations:** 1 Division of Sport and Rehabilitation Medicine, Ulm University Medical Center, Ulm, Germany; 2 Department of University Sports / Workplace Health Management, Ulm University, Ulm, Germany; 3 Ceres - Cologne Center for Ethics, Rights, Economics, and Social Sciences of Health, Unversity of Cologne, Cologne, Germany; 4 Section Health Economics and Health Services Research, Department of Psychiatry II, Ulm University Medical Center, Günzburg, Germany; 5 Institute of General Practice, Ulm University Medical Center, Ulm, Germany

**Keywords:** Willingness to pay, Health economics, Childhood obesity, Intervention, Health promotion and prevention, Public health

## Abstract

**Background:**

Willingness to Pay (WTP) is an alternative to measure quality-adjusted life years for cost-effectiveness analyses. The aim was to evaluate longitudinal changes and determinants of parental WTP for the prevention of childhood overweight and obesity.

**Methods:**

Longitudinal data from post- (T2) and follow-up (T3) measurements of a school-based health promotion program in Germany. Parental questionnaires included general WTP and the corresponding amount to reduce incidental childhood overweight and obesity by half. Longitudinal differences were examined with the McNemar test for general WTP and the Wilcoxon signed-rank test for the amount of WTP. Regression analyses were conducted to detect determinants.

**Results:**

General parental WTP significantly decreased from 48.9% to 35.8% (*p* < 0.001, *n* = 760). Logistic regression analysis (*n* = 561) showed that parents with a tertiary education level and a positive general WTP at T2, families with a higher monthly household income, and those with abdominally obese children were significant predictors of general WTP at T3. Median amount of WTP at T3 was €20.00 (mean = €27.96 ± 26.90, *n* = 274). Assuming a WTP of €0 for those who were generally not willing to pay or did not answer, resulted in a median amount of WTP at T3 of €0 (m = €8.45, sd = €19.58, *n* = 906). According to linear regression analysis WTP at T2 was the only significant predictor for the amount of WTP at T3 (*p* = 0.000, *n* = 181).

**Conclusions:**

Despite the decline of general WTP, these results are a reflection of the public awareness of the problem and the need for action. Policy makers should recognize this and initiate sustainable public preventive strategies.

**Trial registration:**

DRKS, DRKS00000494. Registered 25 August 2010, https://www.drks.de/drks_web/.

## Background

Childhood overweight and obesity are a growing health problem [1] with about one in five children and adolescences being overweight or obese worldwide [2]. Pediatric overweight and obesity prevalence rates, identified using the Body Mass Index (BMI) [2], as well as abdominal obesity rates [3], defined as a Waist-to-Height-Ratio (WHtR) equal or greater than 0.5 [4], are rising. A higher weight during childhood is associated with cardiovascular disease indicators such as hypertension or metabolic syndrome [5], psychological problems such as anxiety and depression [6, 7], stigma [8] and with lower academic performance [9].

Besides these negative personal consequences, childhood overweight and obesity, and the associated physical inactivity, cause a great financial burden for society. In 2013, costs for physical inactivity were about 53.8 billion Dollar worldwide [10]. Childhood overweight and obesity are related with high pharmaceutical and medical care costs [11] as well as a high usage of healthcare services [12]. In this regard, obese and abdominally obese primary school children have a higher number of sick days and visits to a physician than non-obese children [13]. Furthermore, obese children are likely to become obese adults [14, 15] and therefore the costs are persisting -and probably increasing- into adulthood. The total lifetime costs for obese children and adolescents are estimated to be nearly €150,000 of which about €130,000 account for productivity losses [16].

A great number of preventive and health promoting interventions are aiming at physical inactivity, sedentary behavior and poor nutritional habits in order to prevent or reduce childhood overweight and obesity, but are rarely evaluated in terms of cost-effectiveness [17–19]. However, due to limited financial resources, a deliberate selection of cost-effective interventions is needed. Within cost-effectiveness analyses, Quality Adjusted Life Years (QALYs) are often used as an outcome indicator reflecting the benefit of an intervention from the perspective of the user, but are difficult to apply in the field of prevention as they focus on disease. Therefore, willingness to pay (WTP) can be used as an alternative measure reflecting the monetary value of an intervention from the perspective of the beneficiary [20].

In health economics, WTP measures individual’s monetary values of a change in health status [20, 21] and is frequently estimated by contingent valuation (CV) [20, 22]. However, research on WTP for health promotion, and especially for the reduction of overweight and obesity in children, is scarce. Cawley found in his CV study among New York state residents a mean WTP of $46.41 for a 50% reduction in childhood obesity [23]. Evaluating WTP for the same reduction in childhood obesity in a cross-sectional study, Kesztyüs et al. detected a general WTP for 48.9% and a mean monthly WTP of €23.04 of parents of primary school children in south-western Germany [24]. In particular, parents of overweight and obese children were significantly more often willing to pay [24]. The present study builds upon the findings of Kesztyüs et al. [24] and uses longitudinal data to detect possible changes over time and determinants of WTP.

### Aim

The aim of this study was to evaluate longitudinal changes of general WTP and the amount of WTP for a 50% reduction in the incidence of childhood overweight and obesity in parents of primary school children in the German Federal State of Baden-Württemberg and to evaluate determinants which influence parental WTP.

## Methods

### The Baden-Württemberg-study

The Baden-Württemberg-Study is the outcome evaluation study of the school-based health promotion program “Join the Healthy Boat”, conducted in primary schools throughout the state of Baden-Württemberg in southwestern Germany, a cluster-randomized controlled intervention trial with a waitlist control group. The baseline measurement was conducted in fall 2010 (T1), the post measurement in fall 2011 (T2) and the follow-up measurement in spring 2013 (T3). For the present study, the post and follow-up measurements T2 and T3 were considered. Both the intervention and control group could implement the intervention at these stages of the study. Approval from the Ethics Committee of Ulm University was obtained. The study was registered in the German Clinical Trials Register (DRKS), Freiburg University, Germany (DRKS-ID: DRKS00000494). Detailed information about the trial is described in Dreyhaupt et al. [25].

### The “Join the Healthy Boat” intervention

The aim of the ongoing “Join the Healthy Boat” intervention is to increase a healthy lifestyle in primary school children and prevent them from becoming overweight or obese. The main health topics are the promotion of physical activity and reducing the intake of sugar-sweetened beverages as well as the consumption of screen media. The intervention materials were developed for teachers and comply with the national education plan. They can be implemented in the curriculum, no extra lessons or external persona are required. Not only materials for children are available, but also materials for parents, e.g. family homework. More information concerning the intervention can be found elsewhere [25].

### Data collection

Written informed consent from parents was obtained prior to data collection. Children’s anthropometrics were taken by trained staff, while information about parental health and lifestyle characteristics were assessed using questionnaires and, if possible, separately for mothers and fathers. Socio-economic variables and all questions about general WTP as well as the amount of WTP were also assessed via questionnaires.

### Anthropometrics

Anthropometric measurements of the children were executed by trained staff according to the International Society for the Advancement of Kinanthropometry Standards [26]. Children’s weight was measured with calibrated flat scales and their height using mobile stadiometers (both by Seca® Company, Germany). The Body Mass Index (BMI) was calculated as weight in kilogram divided by height in m^2^. The BMI for children was converted to BMI percentiles, controlling for age and gender using German reference data, with the 90th percentile as overweight and the 97th percentile as obese [27]. Waist circumference (WC) was measured in centimetres with a metal tape (Lufkin® Industries Inc., Texas, USA) exactly between the iliac crest and the border of the lowest rib. The mean of two WC measurements was used and if the difference between them was greater than 1 cm, a third measurement was conducted. The Waist-to-Height-Ratio (WHtR) was calculated by the quotient of WC and height in centimetres and a WHtR ≥0.5 defined as abdominal obese [28].

Self-reported data of parental weight, height, and WC were used to calculate BMI and WHtR. A BMI ≥ 25 was defined as overweight and a BMI ≥ 30 as obese due to WHO standards [29], and a WHtR ≥0.5 as abdominal obese [28].

### Socioeconomic variables

The family education level was defined according to the CASMIN (Comparative Analysis of Social Mobility in Industrial Nations) classification [30] using the highest level of two parents or the level of a single parent. This was dichotomized into a tertiary vs. a secondary and primary level. Single parenthood and monthly household income was assessed, the latter categorized in low (< 2250€), middle (2250€ - 4000€), and high (≥ 4000€). Migration background was assumed if at least one parent mainly spoke a foreign language during the child’s first year of life or at least one parent was born abroad.

### Parental health and lifestyle characteristics

Parents were asked about their health awareness level on a four point rating scale (“very high”, “high”, “little”, “very little”) which then was dichotomized into a high and low level. Parents were asked about the importance of being thin for being attractive on a four point rating scale (“not important at all”, “not important”, “important”, “very important”), which was dichotomized into “not important” vs. “important”. Parents were also asked if they considered their child as too corpulent or too thin on a five point rating scale. The answers were dichotomized in “very corpulent” and “a bit corpulent” on the one side, and “neither/nor”, “a bit thin”, and “very thin” on the other side. Smoking status was dichotomized, with current smokers vs. non-smokers/ex-smokers.

### Willingness to pay

WTP assesses monetary valuations of changes in health by presenting hypothetical scenarios about a certain change in health or an intervention [31, 32]. Individuals’ monetary values are frequently assessed by CV which is measuring individuals’ stated preference by asking them how much money they are willing to pay for the change in health or an intervention [32]. CV is a method assessing WTP in different ways, such as structured telephone interviews or mail surveys and by asking a series of questions narrowing down the bounds of the WTP (so called double-bounded model) or by using an open-ended question [23].

The part of the questionnaire on WTP began with some general information about overweight and obesity, it’s prevalence and it’s health care costs. Parents were asked to indicate whether they thought overweight and obesity were serious public health problems (“yes”, “no”). Afterwards, they were told to imagine a preventive measure reducing the incidence of childhood overweight and obesity by half. The second question was, if they were in general willing to pay for this preventive measure (general WTP; “yes”, “no”). The parents who answered “yes” were asked to indicate the amount of money they were willing to pay for this measure per month (amount of WTP). For T2, 12 answer categories were provided: 1) €1–5; 2) €6–10; 3) €11–20; 4) €21–30; 5) €31–50; 6) €51–75; 7) €76–100; 8) €101–150; 9) €151–200; 10) €201–300; 11) €301–500; 12) > than 500. For T3, an open-ended question was used. For the comparison of the amount of WTP between T2 and T3, data of T3 were converted into the categories of T2.

### Participants

**Fig. 1:**
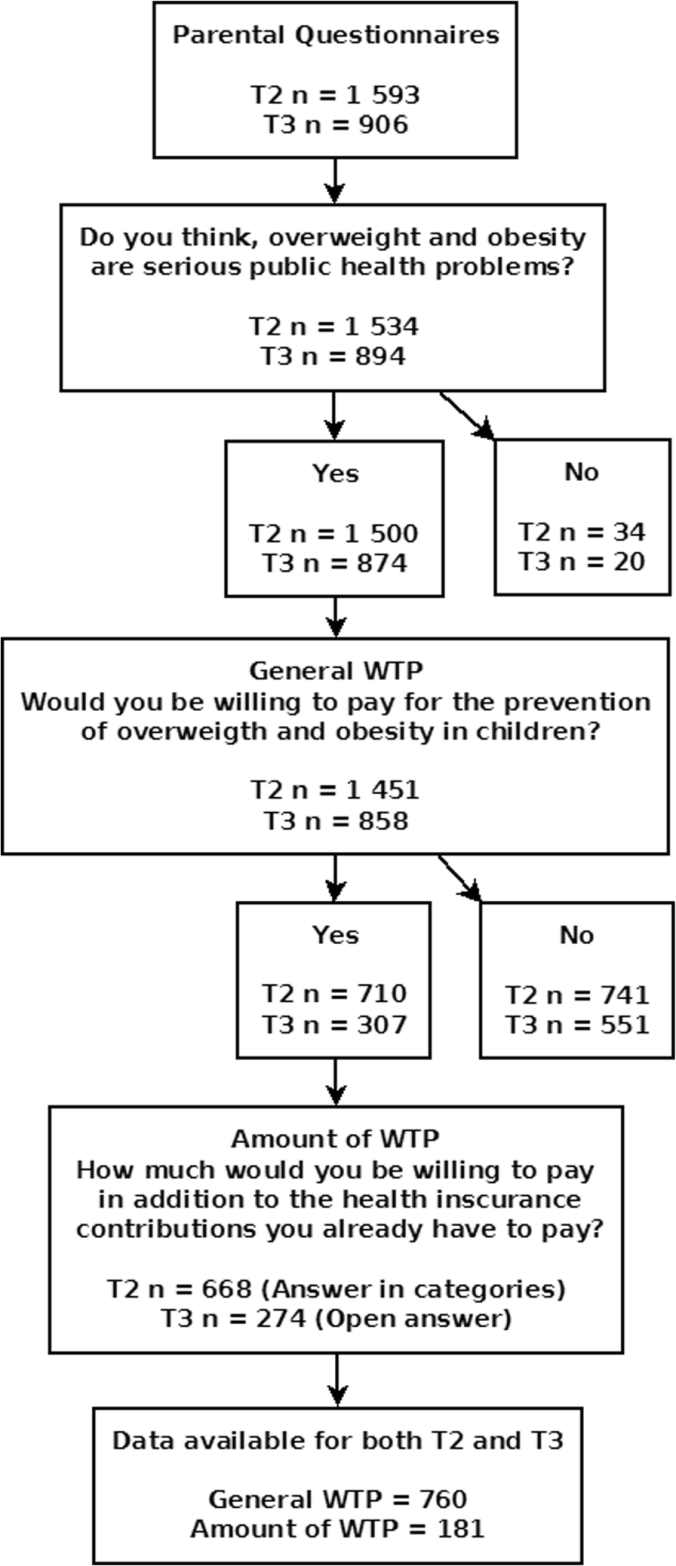
Flow chart showing the respective underlying numbers of datasets available for analyses of the parental willingness to pay (WTP)

For the post measurement, 1829 children participated in anthropometric measurements with available data from 1593 parental questionnaires. For the follow-up measurement, 1043 children participated in anthropometric assessment and 906 parents provided questionnaires. An overview of the underlying numbers of datasets in the different stages of the study are shown in Fig. 1.

### Missing data

Missing data are a commonly occurring problem in observational studies and can lead to bias [33]. Therefore, data with complete datasets were compared to those with missing data to detect possible differences.

### Statistical analyses

Differences in parental and child characteristics between those parents who were in general willing to pay and those who were not willing to pay at T3 were analyzed with Fisher’s exact test for categorical data and Mann-Whitney-*U* test for continuous data at T2. To analyze if parents differed significantly in their answers between T2 and T3, the McNemar test for the general WTP and the Wilcoxon signed-rank test for their amount of WTP were conducted. After calculating a generalized linear mixed model to test potential clustering of data in schools, a logistic regression analysis was executed for the general WTP at T3. As potential explanatory variables, variables assessed at T2 from Table 1 were included according to their significant association with the outcome variable and their relevance of content. For the amount of WTP, a linear regression analysis following the same strategy was conducted for parents who stated a positive general WTP. In order to investigate possible differences between participants with and without missing values for the logistic regression analyses for general WTP at T3, Fisher’s exact test for categorical data and Mann-Whitney-*U* test for continuous data were calculated. To account for clustering in schools, R Release 3.2.3 for Windows (www.cran.r-project.org) was used. All remaining analyses were executed with SPSS 21 (SPSS Inc. Chicago, IL, USA). The significance level for two-sided tests was α = 0.05.

**Table 1:** Participants’ characteristics at T2 for the parental general willingness to pay (WTP) at T3

	**Missing values** **n**	**WTP Yes** ***n*** **= 307**	**WTP No** ***n*** **= 551**
**Parental characteristics**			
Age (mother), m (sd)	79	38.8 (4.9)	38.7 (4.7)
Age (father), m (sd)	118	41.7 (5.8)	41.8 (5.6)
Maternal overweight, n (%)	123	86 (33.3)	141 (29.6)
Paternal overweight, n (%)	182	139 (57.7)	260 (59.8)
Maternal obesity, n (%)	123	37 (14.3)	39 (8.2)*
Paternal obesity, n (%)	182	32 (13.3)	61 (14.0)
Maternal WHtR ≥0.5, n (%)	425	76 (50.3)	145 (51.4)
Paternal WHtR ≥0.5, n (%)	463	96 (69.1)	178 (69.5)
Considering overweight and obesity as a problem, n (%)	58	285 (99.3)	502 (97.9)
Importance of being thin for being attractive (at least one parent), n (%)	68	161 (56.9)	292 (57.6)
Considering child too corpulent (at least one parent), n (%)	39	34 (11.7)	34 (6.4)*
High level of maternal health awareness, n (%)	71	164 (59.4)	324 (63.4)
High level of paternal health awareness, n (%)	147	104 (40.6)	200 (44.0)
Smoking (mother), n (%)	59	57 (20.2)	79 (15.3)
Smoking (father), n (%)	128	68 (26.2)	129 (27.4)
Tertiary family education level, n (%)	64	110 (39.3)	153 (29.8)**
Monthly household income	168		**
< 2250€, n (%)		48 (18.9)	116 (26.6)
2250€ - < 4000€, n (%)		128 (50.4)	240 (55.0)
≥ 4000€, n (%)		78 (30.7)	80 (18.3)
Single parent, n (%)	45	32 (11.0)	60 (11.5)
General WTP yes at T2, n (%)	98	203 (75.2)	181 (36.9)***
**Child characteristics**			
Intervention participant, n (%)	0	154 (50.2)	305 (55.4)
Age, m (sd)	0	8.1 (0.6)	8.0 (0.6)
Boys, n (%)	0	160 (52.1)	267 (48.5)
Migration background, n (%)	73	69 (24.3)	132 (26.3)
Overweight, n (%) Kromeier	16	34 (11.2)	35 (6.5)*
Obesity, n (%) Kromeier	16	18 (5.9)	9 (1.7)**
WHtR ≥0.5, n (%) / Abdominal obesity, n (%)	16	35 (11.5)	31 (5.8)**

*WHtR* waist-to-height-ratio, *WTP* willingness to pay; *p < 0.05, ** *p* < 0.01, *** *p* < 0.001

## Results

Of 894 parents at T3, 97.8% (*n* = 874) indicated overweight and obesity as serious public health problems, without significant differences for parents of overweight or obese children and the others. In the following, results for general WTP, amount of WTP and missing data are shown separately.

### General willingness to pay

At T2 (*n* = 1451), 48.9% of the parents were in general willing to pay (*n* = 710). For the follow-up measurement T3, valid data from 858 parents on their general WTP were available, with 307 (35.8%) declaring their willingness to pay. Characteristics of parents and children are shown in Table 1 separately according to the parental general WTP. Given that missing values occurred and varied per item, the numbers used for the calculation of significant differences deviate from 307 respectively 551 for WTP at T3. For example, the valid cases for paternal WHtR are 307 + 551–463 = 395 (WTP yes + WTP no – missing values). In those 395 valid cases, there was no statistical significant difference concerning WTP in the percentage of fathers with a WHtR ≥0.5 (69.1% vs. 69.5%). Parents of overweight, obese and abdominally obese children, parents who considered their child as too corpulent, obese mothers, and families with a tertiary family education level and a higher household income were significantly more often willing to pay (*p* < 0.05). Parents who were in general willing to pay at T2, were significantly more often willing to pay at T3 (*p* < 0.001).

**Fig. 2:**
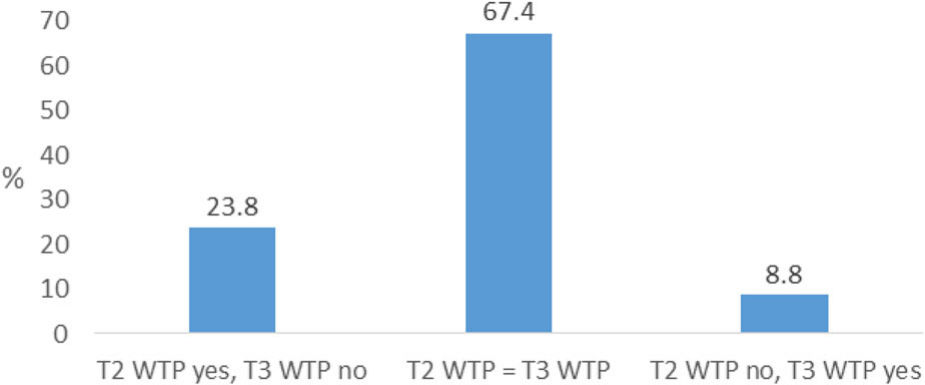
Changes in general willingness to pay (WTP) over time (*n* = 760)

The general WTP was significantly lower at T3 (35.8%) than at T2 (48.9%), χ^2^ = 51.49, *p* < 0.001, *n* = 760. Details of these changes are shown in Fig. 2. Of the 760 parents with valid data, nearly three times more parents were willing to pay at T2, but not at T3 (23.8%) than the other way around (8.8%). Of the parents having the same general WTP at T2 and T3 (67.4%), 39.6% were willing to pay and 60.4% were not willing to pay at both time points.

Since there were no differences between a generalized linear mixed model and an ordinary logistic model, the latter is reported. This logistic regression analysis for the general WTP at T3 is shown in Table 2 (*n* = 561, Nagelkerkes R^2^ = .237). Parents with a tertiary education level and a general WTP at T2, families with a higher monthly household income, and abdominally obese children were significant predictors of general WTP. Maternal obesity and children with migration background were no significant predictors, but remained in the model because they were significant in Table 1 respectively relevant factors in other studies. No significant results for intervention participants were found, therefore this variable was excluded from the regression analyses.

**Table 2:** Adjusted Odds ratios (OR) for the general willingness to pay (WTP) at T3

***n* = 561**	**OR**	***p*-value**	**95% CI**
Maternal obesity	1.84	0.060	0.98–3.48
Tertiary education level	1.53	0.049	1.00–2.34
Monthly household income			
< 2250€		*Reference*	
2250€ - < 4000€	1.26	0.396	0.74–2.14
≥ 4000€	1.93	0.042	1.03–3.63
General WTP at T2	4.80	< 0.001	3.21–7.19
Abdominal obesity child	3.92	< 0.001	1.78–8.66
Migration	0.69	0.105	0.44–1.08

*CI* confidence interval, *OR* odds ratio, *WTP* willingness to pay; R^2^ = .237 (Nagelkerke)

### Amount of willingness to pay

The amount of WTP at T2 had a median of the answer category 3 (11–20€) and an adjusted mean of €23.04 (99% CI [22.45, 23.75], *n* = 710). Assuming a WTP of €0 for those who did not respond and those who were not willing to pay in general, a mean of €10.27 (*n* = 1593) was given [24].

**Fig. 3:**
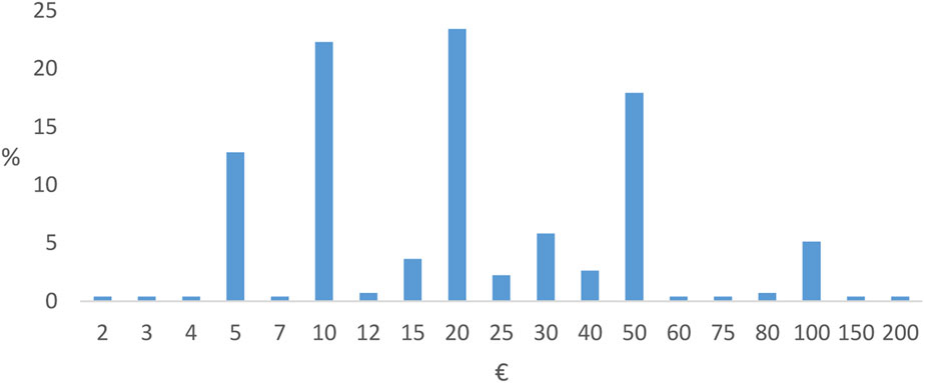
Distribution of the amount of willingness to pay (WTP) per month at T3 (*n* = 274)

Figure 3 shows the amount of WTP per month at T3. Of the parents who were in general willing to pay (*n* = 307, 35.8%), 89.25% (*n* = 274) stated their amount of WTP with a median of €20.00 (m = €27.96, sd = €26.90). Assuming a WTP of €0 for those who did not answer the question or those who were not willing to pay in general, the amount of WTP with a median of €0 (m = €8.45, sd = €19.58, *n* = 906) was given.

**Fig. 4:**
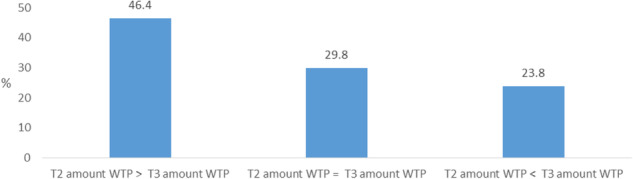
Changes in amount of willingness to pay (WTP) over time (*n* = 181)

The distribution of the amount of WTP differed significantly between T2 and T3 for parents who were in general willing to pay and for whom data for the amount of WTP of both T2 and T3 were available (z = − 2.133, *p* = .033, *n* = 181). The changes in WTP between T2 and T3 are visualized in Fig. 4, with nearly double the number of parents indicating a lower amount of WTP at T3 than at T2 (46.4%) than the other way around (23.8%). Nonetheless, the mean amount of WTP at T3 was higher than at T2 (mean = €27.96, 99% CI [23.74; 32.17], *n* = 274 vs. €23.04, 99% CI [22.45, 23.75], *n* = 710).

The linear regression analyses for the amount of WTP (*n* = 181) showed significant results only for the amount of WTP at T2 (*p* < 0.001; CI 2.58–6.89, R^2^ = .095), increasing the average amount of WTP by €4.74. No other variables were found to be significant predictors of the amount of WTP.

### Missing data

Participating children with missing data were significantly more likely to have a migration background and mothers who were smokers (*p* < 0.01); additionally, parents were significantly more often younger (*p* < 0.01) and also more frequently single parents (*p* < 0.001). Families with missing data had significantly lower monthly household incomes (*p* < 0.001).

## Discussion

To end childhood obesity, the WHO demands a holistic approach where policies across different sectors address health systematically [34]. Beneficial effects of early childhood interventions occur on health and economical levels and can have an influence on the entire family, even years after an intervention [19]. The need for interventions to reduce childhood overweight and obesity requires an assessment of their cost-effectiveness in order to make allocative decisions on which interventions should be promoted. To compare the costs of preventive and health promoting strategies, WTP is a suitable alternative to QALY. However, WTP studies are scarce and, to the authors’ knowledge, no longitudinal WTP study has been carried out for the prevention of childhood obesity before. Therefore, the present study assessed longitudinal changes of general WTP and amount of WTP for a 50% reduction of the incidence of childhood overweight and obesity in parents of primary school children, and also evaluated determinants of WTP.

In the present study, nearly all parents indicated overweight and obesity as serious public health problems, with higher (but not significantly) rates for parents who were in general willing to pay. This implies great awareness of the problem. Cawley [23] found, in almost all of his assessed surveys on childhood obesity, that at least two-third of the respondents indicated childhood obesity as a major problem which was associated with a higher WTP. In addition, the German public has been found to express great support for the prevention of obesity [35].

The general WTP decreased over time, with about every second parent in general willing to pay at T2 but only about every third parent at T3. Reasons for the fact that more than half of the parents were not willing to pay in general and the decrease of the general WTP, could be the German health insurance system in which almost every citizen is insured and the prospect of the “Prevention Strengthening Act”, a government law on prevention efforts, which was implemented in 2015 after a long negotiation period. In this regard, parents might take public services for granted and see no need to pay for them themselves. Additionally, parents’ risk assessment of their children to be overweight or obese in adulthood may be skewed by optimism, as they rate their own child’s risk much lower than that of a typical child [36]. Also, parents could see obesity prevention as a task of the school, e.g. after-school physical education classes. Furthermore, at T3 the children’s change to a secondary school is in sight, which sets the course for further education and employment. Therefore, parents may focus more on school achievements rather than on health subjects.

In parallel to the decreasing of general WTP, a substantial part of parents reduced the amount of their WTP at T3. Narbro and Sjöström argued a WTP of €0 might be a possible protest answer because Swedish participants might not be willing to pay extra money for a treatment that might already be covered by compulsory taxes [37]. However, in contrary to the reductions in the amount of WTP by many parents, the mean amount at T3 with €27.96 was higher than that of T2 with €23.04. Probably those who increased their amount, despite being fewer than those who decreased their amount, must have overcompensated the losses. Different measurement methods could also bias these results.

The mean WTP at T3 of €27.96 per month (€335.52 per year) of the parents who were willing to pay is much higher than the costs for this specific intervention with €24.05 per child per year [38]. Even when considering the average WTP of €8.45 per month (€101.40 per year), assuming a WTP of €0 for parents who were not willing to pay in general or those who didn’t specify an amount of WTP, the WTP exceeded the costs by far.

The scarcity of comparable research of WTP for the reduction of childhood overweight and obesity exacerbates the comparability of the present findings. To the authors knowledge, only one study conducted similar research and reported a WTP of $46.41 per year which outreaches the savings from a 50% reduction of childhood overweight and obesity by far [23]. Furthermore, three studies are known for measuring WTP for obesity reduction in adults in terms of efficacy. Fu et al. [39] reported a WTP for a therapy reducing weight by 5kg in 3 months of $362, while Doyle et al. [40] detected a WTP for obesity pharmacotherapy of $10.49 per month per %-point of weight loss. Narbro and Sjöström [37] assessed a WTP of $3.280 for effective obesity treatment and even reported that participants were willing to borrow money to cover their WTP as it was about twice their monthly income. Other studies found a WTP for a 50% reduction of childhood asthma on household level between $56.48 and $64.84 [41] and a WTP for children’s oral health of €37 per month [42].

### Determinants of willingness to pay

Despite the smaller sample size and marginal differences compared to the cross-sectional data of the parental general WTP [24], the present study shows similar tendencies for determinants of general WTP such as maternal obesity, child’s abdominal obesity and monthly household income.

All of the cross-sectional detected correlates of the amount of WTP [24], except the amount of WTP at T2, were no longer influencing factors at T3. The stability of the WTP amount at T2 supports the idea that the amount of WTP is a fairly stable construct that is not arbitrary and will persist over time.

Several studies support the present findings that families affected by overweight and obesity have a higher WTP. In their studies on obesity prevention and treatment, Fu et al. [39] and Narbro and Sjöström [37] found higher weight to be associated with higher WTP. A higher WTP for participants with a history of the disease was also found in childhood caries prevention studies [42], individual health care costs of older adults [43], and diagnostic technologies mostly for cancer [44]. Therefore, it can be assumed that affected parents are aware of the problems associated with childhood overweight and obesity and take this problem more seriously than their unaffected counterparts.

Studies also confirm the present findings that parents with a higher socio-economic status have a higher WTP. Three studies on the prevention and treatment of obesity found a higher WTP in participants with higher incomes [23] or higher incomes and higher educational levels [37, 39]. In their study on WTP for personalized nutrition Fischer et al. [45] found that a higher income was associated with a higher WTP. Higher income and higher educational level was associated with a higher WTP for childhood caries prevention [42] and for diagnostic technologies for mostly cancer [44]. This is plausible as a high education level seems to be linked to knowledge and awareness of health issues and the availability of money seems to be related to a greater investment capacity.

### Strengths and limitations

The first strength of the present study is its large sample size. The large number of participants and collected data allows to control for several co-variables. Second, data collection in the entire third largest federal state with the third highest population density in Germany provides data for different living conditions of different family constellations. Third, children’s anthropometrics were assessed by trained staff according to standardized protocols. Fourth, to the authors’ knowledge, this is the first study to investigate longitudinal changes in parental WTP for the reduction of childhood overweight and obesity and therefore presents important results. Fifth, the CV method used for the present study has a face validity with a precise description and detailed information to enable participants to make an informed decision [46]. Lastly, pre-tests assured appropriateness of the provided information of the WTP questions and participants’ understanding of the context and therefore minimize bias [21, 46].

However, some limitations have to be mentioned when interpreting the results. First, the present study was carried out primarily as an effectiveness study with the WTP study piggybacked. For organizational reasons, questionnaire surveys were carried out. If the study would have been conducted for health economic purposes only, direct interviews with a double-bounded model would have been recommended [21, 23]. Second, WTP is a hypothetical construct and therefore the assessed WTP can deviate from the real WTP [20, 39]. Because no real financial implications for the participants exist [47], they may misjudge the scenario provided and may overestimate their WTP [42] or their risk [47]. To limit this bias, pre-tests were executed. Additionally, other researchers question the importance of hypothetical bias [48]. Third, two different measurements of the amount of WTP were used (open vs. closed answers), which makes a comparison more complicated and could be the reason for conflicting findings. It is not clear which elicitation method provides most accurate estimate of the WTP [44] and therefore, two measurements were executed to evaluate their advantages and appropriateness for the present study. For the closed-ended payment scales range bias [21], framing effects [49] and bias due to the design of the payment scales can occur [50]. A higher mean WTP was found for a scale providing up to £1000 in comparison to one providing up to £100 [51] and for asking monthly WTP in comparison to yearly WTP [49]. On the other hand, open-ended questions tend to be more imprecise due to a great variation in results [31] and the prominence effect may bias results as respondents tend to provide prominent numbers such as 1, 2, 5, 10, 20, 50 etc. [52]. Fourth, selection and sampling bias may affect the results [21]. Parents are not representative for the entire population, they may tend to be more interested in the subject and more willing to pay than people without children or with children at a different age. However, even if the amount of WTP is overestimated, it is presumably still higher than the costs of the intervention, which is more than four times higher [38]. Finally, the missing data in the present study can be seen as selection bias and are a common problem in epidemiological studies. However, except for the household income, no significant differences in the WTP for those with missing values compared to those with complete data were found.

## Conclusions

Nearly all participating parents rated childhood overweight and obesity as a serious public health problem. Between half and one third of the parents were in general willing to pay for the prevention of childhood overweight and obesity. Families affected by overweight and obesity and those with a higher socioeconomic status were more often willing to pay. The amount of WTP exceeded the costs of the preventive program by far. In sum, despite the decline in general WTP, these are all reflections of the public awareness of the problem and the need for action. Policy makers should be aware of this and translate it into public preventive strategies. Preferably, health promotion and obesity prevention should be sustainable and therefore integrated into the curriculum of school children and even into teacher training. This would be in line with requirements of the WHO asking for a whole-of government approach and the “health-in-all-policies”.

## Data Availability

The datasets generated and analysed during the current study are not publicly available due to data protection but are available from the Institute of Epidemiology and Medical Biometry, Ulm University, on reasonable request.

## Abbreviations

BMI: Body mass index
CASMIN: Comparative analysis of social mobility in industrial nations
CV: Contingent valuation
DRKS: German clinical trials register
OR: Odds ratio
QALY: Quality adjusted life years
WC: Waist circumference
WHtR: Waist-to-height-ratio
WHO: World Health Organization
WTP: Willingness to pay
